# The Hospital Problem

**Published:** 1890-03-15

**Authors:** Henry C. Burdett

**Affiliations:** Deputy Chairman of The Hospitals Association


### THE HOSPITAL PROBLEM.

By Mr. Henry C. Burdett, Deputy Chairman of The Hospitals Association.

(*Continued from page* 354.)

### II.—WHAT IS WANTED.

A study of the questions arising out of the figures given last week extending over a period of a quarter of a century forces me to the conclusion that before any Royal Commission is appointed it is desirable that some further time should be given to the managers of the voluntary hospitals to carefully review the situation, and to inquire into the facts which have been brought to light through the publication of the figures contained in the last edition of the *Hospital Annual* and in those which will be published in the new edition of the *Annual* next month. Until the managers, *i.e.*, those people who are immediately concerned in clearing up the differences proved to exist at the present time in the case of the various hospitals throughout the country, have examined the figures for themselves, and by careful inquiry arrived at a definite conclusion as to the causes which create the differences, and the extent to which they may be lessened by a modification of system or a stricter enforcement of economy, it would, in my opinion, be a mistake to appoint a Royal Commission. I make this statement as one who, for many years, has advocated the appointment of such a Commission, because I am conscious that the mere demand for such an inquiry has produced, and is producing, important results. There is evidence of continuous progress all along the line, and I believe that there are no members of the community more anxious to arrive at the truth and to introduce changes which will secure the maximum of economy combined with efficiency, than the large majority of the hospital managers themselves.

What we ought to endeavour to secure at once is the passing of a short Act through Parliament to assist the managers of reputable charities by enacting—

(1) That no one shall have the power to open a building, wherever they please, and whether it is wanted or not, for the treatment of patients—too frequently misnamed a hospital—unless or until they have satisfied the authorities of—

(*a*) Their *bona fides*; (*b*) the necessity for such an establishment, and the suitability of the site selected: and (*c*) the competency of the promoters to provide the necessary funds, and to secure their proper administration.

These points being determined to the satisfaction say of the County Council of the district, or, if it is preferred, of some central body like the Local Government Board, a licence or permit should be issued to the promoters, and they should then be left free to manage the institution in the ordinary way.

(2) That every hospital and all kindred charities supported by voluntary contributions shall publish annually a proper statement of accounts, including the acknowledgment of every sum received. A schedule of the Act should contain the forms in which these accounts should be kept. The Act should provide that copies of these accounts shall be sent to the County

Council, or central authority as may be deemed best. It is shown later on how these results can best be secured, and the most suitable form of account for hospital purposes which can be adopted.

A short Act embodying these two main provisions would effect all, or nearly all, the reforms which are necessary. The first provision would protect the reputable and well-managed institutions from the discredit and loss resulting at the present time from the establishment of useless and unnecessary institutions which, from their very nature, are compelled to expend large sums of money in the endeavour to raise funds from the charitable at the expense of much-needed and deserving institutions of standing and repute. In other words, if such a law were passed it would at once stop the wasteful and wanton extravagance of a certain class of people who declare that it is nobody's concern that they should expend £300 in advertising and appeals to enable them to raise £400, leaving only £100, or 5s. in the pound, available for the promotion of the objects of the particular institution which they have determined to carry on by hook or by crook. This is not an exaggerated statement, as it has been frequently admitted that much money has been raised in this wasteful and extravagant way.

The second provision would prevent fraud of various kinds. In the course of a long experience I have been asked to serve on committees of investigation and inquiry where the management of an institution has fallen into bad odour. The most common form of fraud is due to the fact that the accounts published in the Reports do not represent the true facts, because a Building Account, a Suspense Account, or some other device has been resorted to, with the view of concealing not only the amount of money received from the public, but the cost at which that money has been raised. Another form of fraud is traceable to the habit of not putting a date upon the counterfoils of the receipt books. In one case where some thousands of pounds had been misappropriated, it was found that, although all the money received was acknowledged in the Report, the sum brought to account was always months, and sometimes even a year, behind. The figures in every account published should be made to agree with the amounts given in detail in the Annual Report, and the public are justified in viewing with suspicion every institution where this practice is not strictly adhered to.

*(To be continued.)*

